# Supplementary material for: How the network properties of shareholders vary with investor type and country
Source: PLoS One. 2019 Aug 23;14(8):e0220965. doi: 10.1371/journal.pone.0220965 (PMC6707624; doi:10.1371/journal.pone.0220965)
Supplement: S1 Appendix — (PDF) [file pone.0220965.s001.pdf]

Supporting Information
for
Shareholder Networks

Qing Yao[1,2,*], Tim S. Evans[1,2], Kim Christensen[1,2]

1 Centre for Complexity Science, Imperial College London, London, SW7 2AZ, U.K.
2 Blackett Laboratory, Imperial College London, London, SW7 2AZ, U.K.

Appendix

A Network Definition

We will set up the basic notation and definitions of the networks used in this work. We have a set of SHAREHOLDERS $\mathcal{S}$, labelled s_i, who hold shares in one or more COMPANIES, the set $\mathcal{C}$ labelled c_j etc. In addition, each shareholder $s \in \mathcal{S}$ carries a TYPE label $\tau(s) \in \mathcal{T}$ where $\mathcal{T}$ is the set of fifteen different labels as given in the main paper.

It is sometimes convenient to indicate the subset of shareholders of one particular type so we use $\mathcal{S}_\alpha$ to indicate the set of shareholders of type $\tau \in \mathcal{T}$

$$\mathcal{S}_\alpha = \{s | s \in \mathcal{S}, \tau(s) = \alpha\}. \tag{A1}$$

We can use our data to define a CORPORATION-SHAREHOLDER NETWORK, $\mathcal{B}$ in which the set of nodes, $\mathcal{V}_B$, are the union of the set of shareholders and companies, $\mathcal{V} = \mathcal{S} \cup \mathcal{C}$. An edge is present in this network between a shareholder and a company if the shareholder has shares in that company.

In practice our work focusses on a projection of the corporation-shareholder network onto just the shareholder nodes. That is we define the SHAREHOLDER NETWORK $\mathcal{P}$ to have a set of nodes $\mathcal{S}$, the set of shareholders. An edge between two shareholders, say s_i and s_j, exists in this network if both s_i and s_j have invested in the same company (at a level above our threshold). In terms of an adjacency matrix P for this network, we have that

$$P_{s_i s_j} = \begin{cases} 1 & \text{if} \quad \sum_c B_{s_i c} B_{s_j c} > 0 \text{ and } s_i \neq s_j \\ 0 & \text{if} \quad \sum_c B_{s_i c} B_{s_j c} = 0 \text{ or } s_i = s_j \end{cases}. \tag{A2}$$

This ensures the shareholder network $\mathcal{P}$ is a simple network.

B Betweenness Centrality

A WALK is a sequence of vertices in which each node is connected by an edge to the next node in the sequence. A PATH is a walk in which no node appears twice. The LENGTH OF THE PATH is the number of vertices minus one, i.e. the number of edges traversed as one moves through the sequence of vertices.

For many centrality measures we consider the shortest path from an initial source node s and ending with a target node t. The number of shortest paths from s to t is denoted by σ_{st} as there can be more than one path of the same length between any pair of vertices. Given these shortest paths, we define $\sigma_{st}(v)$ to be the number of these shortest paths which pass through some v other than s or t. Then, the BETWEENNESS [1, 3] $b(v)$ of a node $v \in \mathcal{S}$ is defined to be

$$b(v) = \sum_{s \neq v \neq t \in V} \frac{\sigma_{st}(v)}{\sigma_{st}}. \tag{A3}$$

C Closeness centrality

We will define CLOSENESS CENTRALITY $c(v)$ [2, 3] of a vertex v to be

$$c(v) = \frac{n-1}{\sum_{u=1}^{n-1} d(u,v)}, \tag{A4}$$

where $d(u,v)$ is the shortest path distance between u and v and n is the number nodes in the component connected to node n.

C.1 Estimating Closeness

Consider first a general random graph, that is, one with a specific degree distribution but otherwise unconstrained, working in large sparse graph regime, $N \to \infty$, $\langle k \rangle \sim O(1)$. This type of configuration model graph can be constructed using edge rewiring. Suppose we start at a node of degree k. Then we might estimate that the number of nodes ℓ steps away from our starting node is

$$n_\ell \;=\; \bar{z}^{\ell-1} k\,, \qquad \ell \geq 1\,, \tag{A5}$$

where $\bar{z}$ is some effective BRANCHING RATIO. That is we expect each node we arrive at ℓ steps away from our starting node, in some breadth first search out from the initial node, to be connected to an average of $\bar{z}$ new vertices which are then $(\ell+1)$ steps away. The approximation here is that all nodes look the same as they must in a true random graph. The exception is the first node where we know that that has k neighbours if that node has degree k. However we note that statistically, all we are really saying in this approximation is that for most networks, taking a few steps is sufficient to allow us to sample any part of the network so statistically many networks will appear to be homogeneous on larger scales.

If we are being more precise, for a random graph near its phase transition, where we can assume a tree like structure, we know that $\bar{z}$ will be the average degree of a neighbouring node minus one — we arrive on one edge going into a neighbour, leave on the remaining edges. Because the current degree of a neighbour $\sum_k k\frac{kp(k)}{\langle k \rangle} = \frac{\langle k^2 \rangle}{\langle k \rangle}$. So

$$\bar{z} \;=\; \frac{\langle k^2 \rangle}{\langle k \rangle} - 1\,. \tag{A6}$$

However, for any given large network, we do not need to assume (A6) is true, merely that there is some effective branching ratio such that (A5) still works well.

To estimate closeness, we first estimate the maximum distance $\ell_{\max}$ by demanding that the total number of nodes connected to our starting node is the number in the Largest Connected Component N_{LCC} as we assume we are studying nodes in this component. This may be estimated as

$$N_{\mathrm{LCC}} \;\approx\; \sum_{\ell=0}^{\ell_{\max}} n_\ell = 1 + k\frac{(\bar{z}^{\ell_{\max}} - 1)}{(\bar{z}-1)} \tag{A7}$$

Rearranging for $N_{LCC} \gg 1$, we find that

$$\ell_{\max}(k) \;\approx\; \frac{\ln(1 + N_{\mathrm{LCC}}(\bar{z}-1)/k)}{\ln(\bar{z})}\,. \tag{A8}$$

Not surprisingly, if you start from a high degree node, a high k, your first step will reveal far more of the network and so take you closer to the remaining parts. Thus the maximum distance in a random graph drops as the degree k of the node increases.

Now we can use this to find the closeness $c(v)$ of a node v since this is defined to be the inverse of FARNESS, $f(v)$, the average distance from a node to all other nodes. For the random graphs, or graphs which appear homogeneous on larger scales, we can estimate farness using (A5) as

$$f(v) \;=\; \frac{1}{(N_{\mathrm{LCC}}-1)}\sum_{\ell=1}^{\ell_v} \ell n_\ell \approx \frac{1}{N_{\mathrm{LCC}}} k_v \left(\frac{(\ell_v+1)\bar{z}^{\ell_v}}{\bar{z}-1} - \frac{\bar{z}^{\ell_v+1}-1}{(\bar{z}-1)^2} \right) \tag{A9}$$

where we have used (A7) and we write $\ell_v = \ell_{\max}(k_v)$ as the largest of the shortest path lengths from vertex v which has degree k_v. Not surprisingly this is dominated by the distance to the further nodes as in the tree they are the dominant contribution. We see that if $(\bar{z}-1) \gg k/N$, i.e. if we are not close to the transition and we have a large N, then this result for farness gives us that $f(v) \approx \ell(v)$ so that

$$f(v) \;\approx\; \frac{\ln(N_{\mathrm{LCC}}(\bar{z}-1)/k_v)}{\ln \bar{z}}\,. \tag{A10}$$

While in this limit a random graph, let alone a real graph, is not a tree, it shows that we should expect the closeness centrality measure to be correlated with the degree of a node. Indeed the prediction is that the inverse closeness (farness) should show a linear dependence on the logarithm of the degree of a node, $\ln(k)$, with a slope that is the inverse of the log of the branching ration minus one, $1/\ln \bar{z}$, that is

$$\frac{1}{c(v)} = f(v) = -\frac{1}{\ln \bar{z}} \ln(k_v) + a\,. \tag{A11}$$

Since this expression is true where we do not have a tree, we do not expect the slope to match a the value of $\bar{z}$ in a random tree (A6). Rather, if we do find a linear relationship for the farness and logarithm of degree, then the slope is a way of defining an effective branching ratio.

D Community detection algorithms

The Louvain algorithm [4] aims to produce a community structure which has a large value of modularity Q where

$$Q = \frac{1}{2m}\sum_{ij}\left[A_{ij} - \frac{k_i k_j}{2m}\right]\delta(c_i c_j)\,. \tag{A12}$$

Here A_{ij} represents the adjacency matrix between nodes i and j; k_i and k_j are the sum of the weights of the edges attached to nodes i and j, respectively; m is the total number of edges in the graph. c_i and c_j are the communities of the nodes. The Louvain algorithm [4] starts each node in an individual community and tries to increase modularity by moving a node into the community of a neighbour. When a local maximum is reached, the communities are used to define a new graph where each node in the new network represents a single community in the previous network, and the process is repeated.

The Infomap community detection is based on the movements of a random walker. The aim is to choose communities which minimise the amount of information needed to record the movement of random walkers between communities. This is done using the map equation:

$$L(M) = q_{\curvearrowleft} H(Q) + \sum_{i=1}^{m} p_{i\circlearrowright} H(P_i), \tag{A13}$$

where M is the modules or partitions of the network and each node is assigned to a module i. $L(M)$ is the description length of the trajectory of a random walker walking along the links of the networks. $q_{i\curvearrowleft}$ and $q_{i\curvearrowright}$ represent that the random walker enters and exits each module i, respectively. For details see Rosvall and Bergstrom [5].

E Comparison of community detection results for largest component of Turkey

If the structure of communities in the data is well established then using two different detection methods should be able to give similar results [6]. After detecting the communities of the graphs using the two algorithms, Louvain [4] and Infomap [5], for two countries, we found that the percentage of nodes whose two communities contain the same nodes is about 75% in Turkey. However, it is noticed that the Louvain method [4] produces a very large community size, while Infomap does not have this large community.

If we look at the largest component, the two different methods are separating this component in different ways, see Figure A1. We can see from Fig A1, most outside parts of the circles are drawn the same shape of nodes in the same colours which means the these nodes are in one community in both methods. In the center of the graphs, that nodes are coloured differently show these square nodes are in same community in Louvain but in different communities in Infomap method. In Table A1 we give out the statistics of the comparison of communities.

		Infomap	Percentage	Louvain	Identical	Ranking of Louvain
1st Largest	Size Community	130	100%	1199		1st
	Types	9		15		
2nd Largest	Size Community	65	100%	93		3rd
	Types	5		5		
3rd Largest	Size Community	58	100%	58	yes	6th
	Types	4		4		
4th Largest	Size Community	58	100%	75		4th
	Types	4		4		
5th Largest	Size Community	56	100%	56	yes	7th
	Types	5		5		
6th Largest	Size Community	51	100%	1199		1st
	Types	5		15		
7th Largest	Size Community	41	100%	41	yes	13th
	Types	4		4		
8th Largest	Size Community	38	100%	132		2nd
	Types	4		6		
9th Largest	Size Community	38	100%	38	yes	15th
	Types	4		4		
10th Largest	Size Community	37	100%	37	yes	17th
	Types	4		4		

Table A1. Table for comparison between algorithms. Table for comparison between the consisting companies in large community for Louvain and Infomap algorithms applied to Turkish network. It is ordered by the community size of the results of Infomap, the percentage 100% means this Infomap community is the subset of Louvain community in this row. The ranking of Louvain reveals the size ranking of this Louvain community.

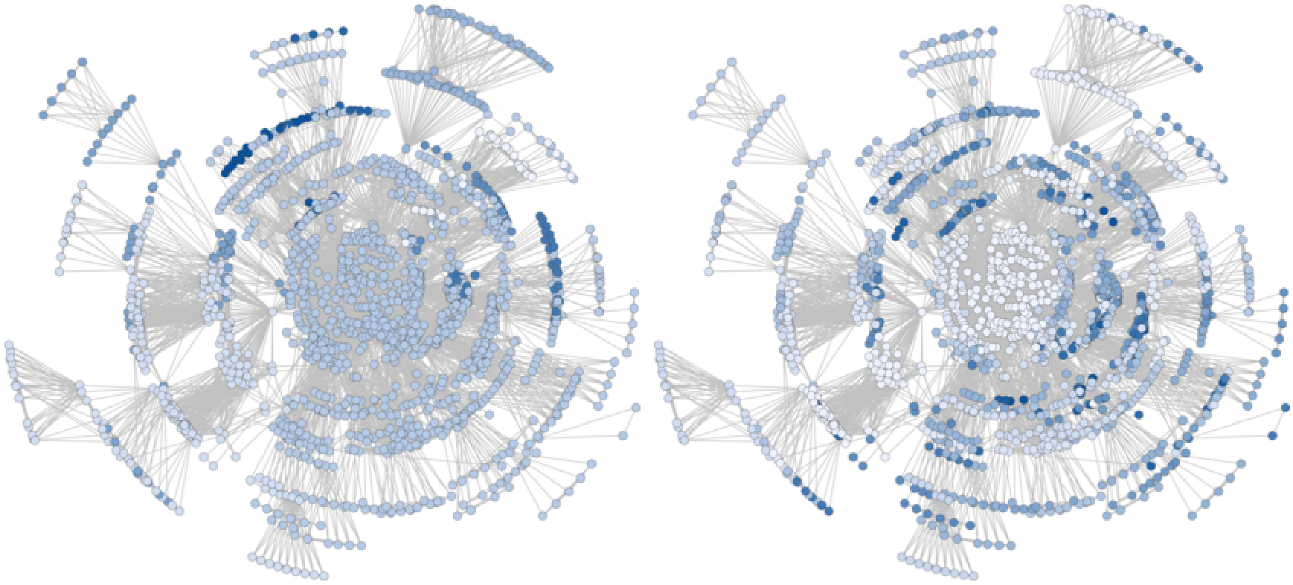

(a) Louvain method **(b) Infomap method**

Figure A1. Comparison between the two detection methods. The left one is for Louvain method and the right one is for Infomap method. The layout style is based on force-directed graph drawing. The number of unique communities for Louvain method is 9 and for Infomap is 124. Each colours represents a community and the colour schemes of the two methods are the same.

F Louvain analysis of Individuals and Industrial in Turkey

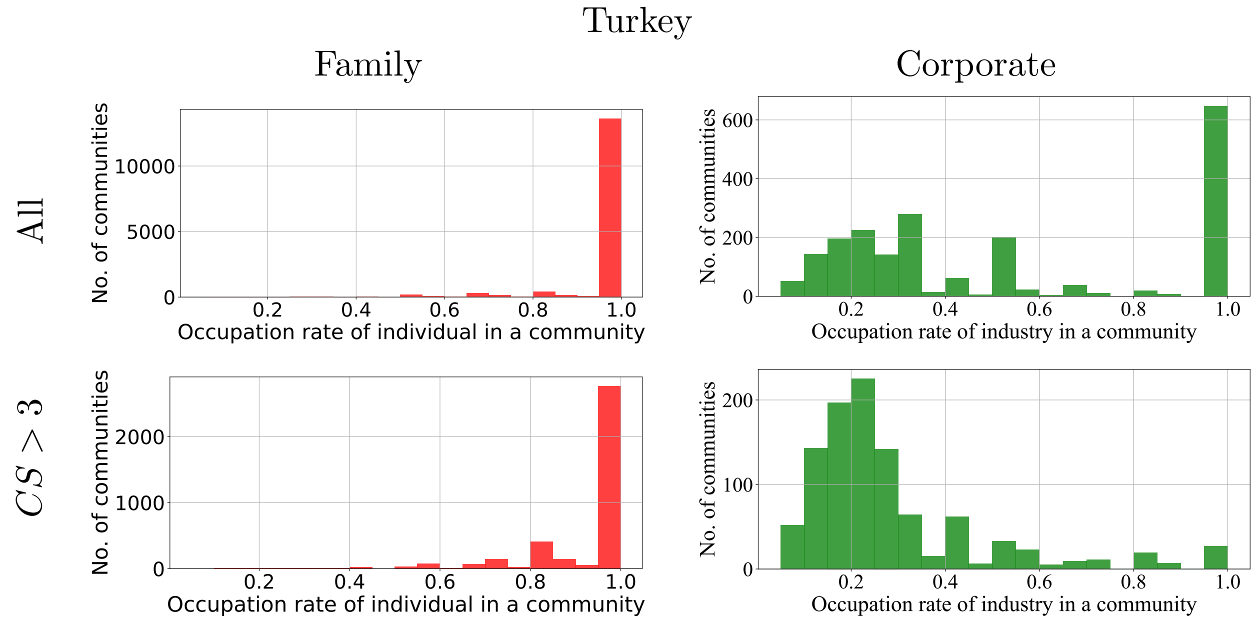

Figure A2. The bar plots for Louvain community analysis. The bar plots of frequency analysis for One or more named individuals or families (upper ones), Corporate company (lower ones) in Turkey: Comparison between the frequencies of percentages of this type of owners within one community. The method used is Louvain. The figures in first row analyses all the community sizes while those in second row excludes small communities(including $CS \geq 3$)

G Update of Data Base

The data of two countries is retrieved from BvD [7], which is updated every year. The total number of known companies in a given year changes. For example, a 4% difference is observed from 2017 to 2018. However, the authors have downloaded the data and done the analysis at different years from 2016, 2017 and 2018. The results described in the main text show no noticeable differences.

References

[1] Freeman LC. Centrality in social networks conceptual clarification. Social Networks. 1978;1(3):215–239.

[2] Bavelas A. Communication patterns in task-oriented groups. The Journal of the Acoustical Society of America. 1950;22(6):725–730.

[3] Newman M. Networks: an introduction. OUP Oxford; 2010.

[4] Blondel VD, Guillaume JL, Lambiotte R, Lefebvre E. Fast unfolding of community hierarchies in large networks. J.Stat.Mech 2008; p. P10008. doi:10.1088/1742-5468/2008/10/P10008.

[5] Rosvall M, Bergstrom CT. Maps of random walks on complex networks reveal community structure. Proceedings of the National Academy of Sciences. 2008;105(4):1118–1123.

[6] Lancichinetti A, Fortunato S, Radicchi F. Benchmark graphs for testing community detection algorithms. Physical Review E. 2008;78(4):046110.

[7] Dijk BV; 2017. Available from: `https://www.bvdinfo.com/en-gb/home`.
